# Detection of *Wolbachia* in *Aedes albopictus* and Their Effects on Chikungunya Virus

**DOI:** 10.4269/ajtmh.16-0516

**Published:** 2017-01-11

**Authors:** Noor Afizah Ahmad, Indra Vythilingam, Yvonne A. L. Lim, Nur Zatil Aqmar M. Zabari, Han Lim Lee

**Affiliations:** 1 Medical Entomology Unit, World Health Organization Collaborating Centre for Vectors, Institute for Medical Research, Kuala Lumpur, Malaysia.; 2 Department of Parasitology, Faculty of Medicine, University of Malaya, Kuala Lumpur, Malaysia.

## Abstract

*Wolbachia*-based vector control strategies have been proposed as a means to augment the currently existing measures for controlling dengue and chikungunya vectors. Prior to utilizing *Wolbachia* as a novel vector control strategy, it is crucial to understand the *Wolbachia*–mosquito interactions. In this study, field surveys were conducted to screen for the infection status of *Wolbachia* in field-collected *Aedes albopictus*. The effects of *Wolbachia* in its native host toward the replication and dissemination of chikungunya virus (CHIKV) was also studied. The prevalence of *Wolbachia*-infected field-collected *Ae. albopictus* was estimated to be 98.6% (*N* = 142) for females and 95.1% (*N* = 102) for males in the population studied. The *Ae. albopictus* were naturally infected with both *w*AlbA and *w*AlbB strains. We also found that the native *Wolbachia* has no impact on CHIKV infection and minimal effect on CHIKV dissemination to secondary organs.

## Introduction

*Aedes aegypti* and *Aedes albopictus* are competent vectors for dengue virus (DENV) and chikungunya virus (CHIKV). The latter also known as Asian tiger mosquito always receive(s) lesser attention as it is considered the bridge vector to *Ae. aegypti*-dominated urban epidemics.^1^ Nonetheless, the Asian tiger mosquitoes can still act as the principle vector in epidemic areas where *Ae. aegypti* is present.^1^,^2^ The worldwide expansion of the geographic range of *Ae. albopictus* makes this invasive vector of human pathogenic viruses a major concern in many locations.^1^ In 2005, *Ae. albopictus* was incriminated as a sole vector responsible for causing chikungunya outbreak of unprecedented magnitude in the Indian Ocean.^3^ The outbreak continued to spread to central Africa,^4^ India,^5^ and then towards Europe,^6^ Asia,^7^–^10^ and North America.^11^ In Malaysia, a nationwide outbreak occurred in 2008, starting in Johor State, which later spread to other states and federal territories affecting about 10,000 people.^9^,^12^,^13^ Phylogenetic analysis of the viral sequence isolates revealed a point mutation of alanine to valine at point 226 (A226V) of the *E1* gene of the polyprotein, enhancing the CHIKV replication and transmission efficacy in *Ae. albopictus*.^14^,^15^

*Wolbachia* species are obligate intracellular bacteria that infect a wide range of insects as well as some species of nematodes, making it the most ubiquitous bacteria yet described.^16^,^17^ *Wolbachia* infection has also been detected in mosquitoes including *Ae. albopictus* but is not found in *Ae. aegypti*. *Wolbachia* are vertically transmitted from infected females to their progeny. *Wolbachia* can alter the reproduction of its host in various ways, one such way is cytoplasmic incompatibility (CI). CI is a form of sterility in which if the same and compatible *Wolbachia* strain is not present in the egg during embryogenesis, embryonic development will be disrupted.^18^,^19^ CI phenomenon gives a reproductive advantage to the infected females, at which they can mate successfully with both infected and uninfected males and hence enhances *Wolbachia* invasion in a population. *Wolbachia* has drawn much attention as some of the *Wolbachia* strains (e.g., *w*MelPop and *w*Mel+wAlbB) have shown to reduce mosquito life span and/or induce pathogen blocking effects on the invertebrate hosts. These effects can substantially reduce the risk of pathogen transmission.^20^,^21^

Nonetheless, the *Wolbachia*-mediated viral blocking effect is not ubiquitous. Unlike the case for transinfected hosts, effect of *Wolbachia* on virus replication in native hosts has been reported to be inconsistent. For instance, the naturally occurring *Wolbachia* of *Aedes notoscriptus* do not induce DENV interference within the native hosts^22^ contrary to the report on *Ae. albopictus* that demonstrated that native *Wolbachia* can limit transmission of DENV.^23^ Another study on *Drosophila* demonstrated that native *Wolbachia* render pathogen resistance toward the RNA viruses in their original hosts.^24^–^26^ The *Wolbachia*-based vector control strategies have taken the form of either population replacement or the incompatible insect technique (IIT) strategy. The population replacement strategy is highly dependent on the ability of the *Wolbachia* to invade and replace the target population with a population that cannot transmit virus.^27^,^28^ On the other hand, the IIT approach involves a continuous inundated release of males carrying an incompatible *Wolbachia* strain with that in the existing mosquito population, to suppress mosquito numbers below a threshold that enables continued virus transmission.^29^,^30^ In this study, we aim to determine the *Wolbachia* infection in field-collected *Ae. albopictus* from different geographical regions. This study is crucial to cater for the scarcity of information on *Wolbachia* infection status in field-collected *Ae. albopictus* population in Malaysia. Furthermore, we also investigated the effects of the naturally occurring *Wolbachia* on the replication of CHIKV in *Ae. albopictus*. These findings will help to facilitate the understanding of the *Wolbachia*–CHIKV–*Ae. albopictus* interaction, which will serve as a platform for the *Wolbachia*-based vector control approach to be conducted in Malaysia.

## Methods

### Mosquito collection.

**Figure 1.**
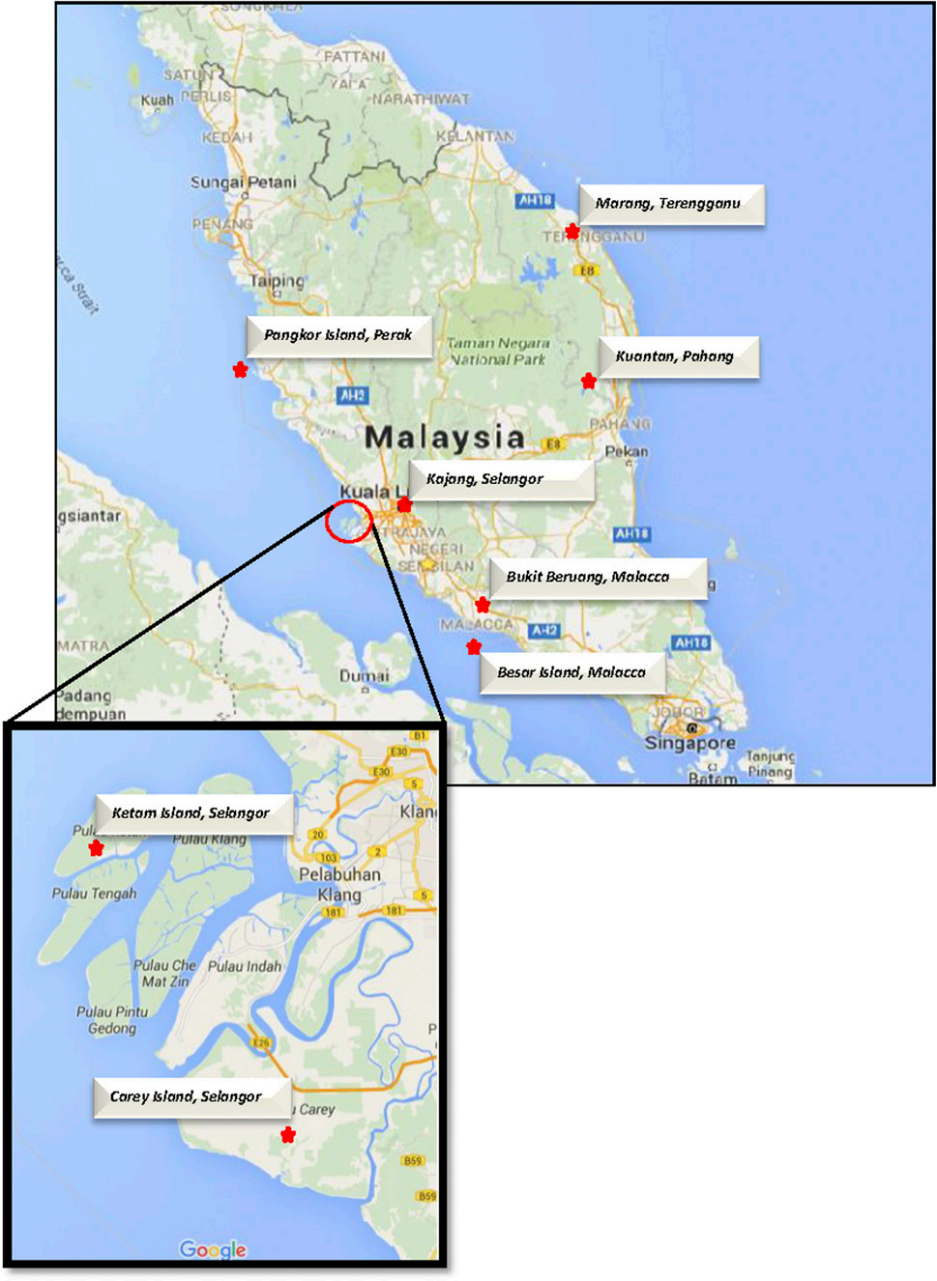
Map of Peninsular Malaysia showing *Aedes albopictus* collection sites. Samples were collected from eight different collection sites (indicated by red stars) from five states in Peninsular Malaysia.

*Aedes albopictus* was collected from eight collection sites from five states in Malaysia as shown in Figure 1

. A minimum of 50 ovitraps were set in each location for 5 days and were at least 150 m apart to minimize the probability of progeny from the same mother. *Aedes* ovitrapping was conducted following the guidelines of Ministry of Health, Malaysia, on ovitrap deployment.^31^ Ovitraps were brought back to the insectarium in the Medical Entomology Unit, Institute for Medical Research (IMR), Kuala Lumpur. Eggs collected were hatched and larvae (L3) recovered were individually identified to species level according to the key by Mahadevan and others.^32^ The identified *Ae. albopictus* larvae from each ovitrap were placed into a plastic container and were supplied with liver powder. Once the larvae reached pupal stage, the plastic containers were placed inside an adult cage (25 × 25 × 50 cm). Mosquito species were again confirmed. Adult mosquitoes were supplied with 10% glucose incorporated with liquid B-complex (Atlantic Laboratories Corp. Ltd, Bangkok, Thailand) and maintained using standard condition of 28°C with 70–80% of relative humidity.

### DNA extraction.

Adults (F0) aged between 7 and 10 days were subjected to DNA extraction. Briefly, 4–10 mosquitoes (males and females) recovered from each ovitrap were killed by placing them in freezer for an hour. The mosquitoes were individually homogenized in 180 μL cell lysis solution (ATL Buffer) and incubated in 20 μL proteinase K at 56°C in water bath for 3 hours. The subsequent procedures were performed according to the QlAamp^®^ DNA Mini Kit protocol (Qiagen^™^, Hilden, Germany).

### Detection of *Wolbachia*.

Multiplex polymerase chain reaction (PCR) was carried out using a temperature profile of 95°C for 15 seconds, 57°C for 30 seconds, and 72°C for 1 minute for 35 cycles using *wsp* primers. Primers used were 328F and 691R for *w*AlbA strain and 183F and 691R for *w*AlbB strain as described by Zhou^33^ (328F, 5′-CCA GCA GAT ACT ATT GCG-3′; 183F, 5′-AAG GAA CCG AAG TTC ATG-3′; 691R, 5′-AAA AAT TAA ACG CTA CTC CA-3′). The PCR mixture contained 5 μL of extracted DNA, 12.5 μL of MyTaq^™^ Mix (Bioline, Taunton, MA), 1 μL of each primer (10 μM), and 4.5 μL of ddH_2_O. Negative and positive controls for the PCR assay were included in each run. The positive control was obtained by screening the adult *Ae. albopictus* (resident strain) using PCR and sequencing of *wsp* gene to confirm that the amplified PCR product obtained was *Wolbachia*. The quality of the extracted DNA was checked using the 12S rRNA primer sets (12SA, 5′-AAA CTA GGA TTA GAT ACC CTA TTA T-3′; 12SB, 5′-AAG AGC GAC GGG CGA TGT GT-3′) to screen samples that were negative for *wsp* primers using the temperature profile of 95°C for 15 seconds for denaturation, 47°C for 30 seconds for annealing and 72°C for 1 minute for extension, conducted for 35 cycles. Samples that were negative for *wsp* primers but positive for 12S RNA primers were scored as uninfected. All the positive PCR products were visualized under 1.5% agarose gel electrophoresis.

### Sequencing of *Wolbachia* endobacterium.

The positive PCR product was purified using QIAquick^®^ Gel Extraction Kit (Qiagen™) before DNA sequencing. A minimum of 10 purified DNA extracts from individuals of each locality were outsourced for sequencing. All sequences were searched against the GenBank nucleotide database using the Basic Local Alignment Search Tool (BLAST^®^) provided by the National Center for Biotechnology Information (http://blast.ncbi.nlm.nih.gov/Blast.cgi). Partial *wsp* gene sequences of *Wolbachia* were aligned using the Clustal-W algorithm and the evolutionary distances of *Wolbachia* isolates from *Ae. albopictus* was constructed using neighbor-joining tree, utilizing Kimura-2P analysis with 1,000 bootstrap replicates in MEGA 6.0 software.^34^ A *Wolbachia* sequence from *Culex quinquefasciatus* was included as an outgroup.

### CHIKV production.

CHIKV (Asian strain) was provided by the Virology Unit, IMR, Kuala Lumpur. The virus was isolated during the outbreak in Bagan Panchor, Perak, in 2006. The CHIKV was maintained in BHK-21 cell lines. Stock virus prepared by freeze-thawing the infected cells once, centrifuging the suspension at 40,000 × *g* and storing the filtered supernatants at 80°C. The infected cells were maintained in Virology Unit, IMR. The titer of the CHIKV stock was determined using the 50% cell culture infectious dose assay.

### Mosquito samples for artificial oral infection.

Mosquitoes from three localities were used for the oral infection experiment. Each locality was chosen to represent one habitat: 1) Besar Island (tourism island), 2) Tenggol Island (remote island), and 3) Bandar Rinching (urban residential area). Females from Besar Island were derived from the same collections used for *Wolbachia* screening. Mosquitoes from Tenggol Island and Bandar Rinching were derived from the existing colony in insectarium. All mosquitoes used for artificial oral infection were F6 generation.

### Tetracycline treatment to clear *Wolbachia*.

Adult mosquitoes were provided with a solution of 0.75 mg/mL tetracycline dissolved in 10% sucrose. After every treatment, 10 randomly selected treated mosquitoes from each generation were tested by PCR for *Wolbachia* infection. Treatment was continued if any randomly tested mosquitoes were positive for *Wolbachia* confirmed via PCR. This treatment was performed up to four generations of mosquitoes. Colonies of *Wolbachia*-free *Ae. albopictus* were maintained for a further two generations without tetracycline before experiments commenced to allow the reestablishment of beneficial microbiota.

### Experimental oral infections.

Experimental oral infection with CHIKV was conducted within an Arthropod Containment Level 2 insectarium. The artificial membrane feeding technique was performed using the Hemotek Feeding Systems (Discovery workshops, Accrington, United Kingdom) housed in an isolation glove box. Human blood used for artificial feeding was sent to Virology Unit, IMR, and confirmed to be negative by neutralization assay for CHIKV antibodies. Blood suspension containing 1:9 of CHIKV (titer 10^7^ plaque-forming units/mL) in human blood was used for artificial feeding. Uninfected samples (control group) were obtained by feeding the mosquitoes with human blood only. A total of 250 adult mosquitoes from each group *Wolbachia* infected (w+) and *Wolbachia*-free (w−) aged 3–5 days that have been starved overnight were subjected to artificial feeding. The blood was presented to the mosquitoes by placing the cups (containing 50 mosquitoes each) below the feeder with the surface of the nylon netting of the cup in contact with the membrane of the feeder. Mosquitoes were allowed to feed for approximately 20–30 minutes. The mosquitoes in cups were cold anesthetized by placing in −20°C freezer for 30 seconds. The mosquitoes were then sorted. All unfed mosquitoes were discarded. Engorged mosquitoes were placed in cups (10 per cup) and kept in incubator at 28°C and humidity of 80% for planned time points (days 0, 1, 2, 3, 5, 7, and 10) postinoculation (PI) studies. At each time point, mosquito samples from at least a single cup were cold anesthetized by placing them in −20°C for 30 seconds, before dissection to remove midguts, salivary glands, and ovaries. Individual mosquito was put on a glass slide. The thorax and head of the mosquito were first removed, followed by dissection of the salivary glands in a drop of saline. The abdomen was then dissected to remove midguts and ovaries in a drop of saline, respectively. Glass slide was replaced for each individual mosquito, and fresh drops of saline were used for each organ examined. It was ensured that the dissecting needles were rinsed in alcohol between each dissection to prevent contamination. A total of four engorged mosquitoes fed with clean human blood at days 0 and 10 PI were kept aside to serve as negative control. For each experimental time point, the infection rate is defined as the number of midguts with detectable virus titer divided by the number of mosquitoes sampled. The dissemination rate was defined as the number of salivary glands with detectable virus titer divided by the number of midguts with detectable virus titer.

### Nucleic acid extraction and quantitative PCR.

Total nucleic acid was extracted from the dissected organs (midguts and salivary glands). Extraction was performed with innuPREP DNA/RNA Mini Kit (Analytik Jena AG, Jena, Germany) that enables the isolation of both RNA and DNA. RNA was used to determine viral load by real-time reverse transcription PCR (RT-PCR), and DNA to check for the presence of *Wolbachia* in ovaries using conventional PCR. For w+ group, individual mosquito that was screened negative for *Wolbachia* (in ovaries) was excluded from the dataset. A minimum number of nine mosquitoes were used for each time point. A standard curve was generated using 10-fold serial dilutions of RNA synthetic transcript with known copy number.

### Determination of limit of detection.

RNA template of known concentration was diluted using six 10-fold serial dilutions. Each concentration was run in triplicate for a total of three runs. Dilutions of CHIKV RNA ranged from 2.4 × 10^6^ RNA copies to 2.4 × 10^1^. The linear range was established with acceptance criteria of *R*^2^ > 0.98 with an efficiency of > 90%. The limit of detection (LOD) was defined as the lowest concentration of viral RNA that can be detected in ≥ 95% of nine replicates.

### Statistical analysis.

GraphPad Prism version 7.00 for Windows (GraphPad Software, La Jolla, CA; www.graphpad.com) was used to construct graphs. All statistical analyses were conducted using the IBM SPSS Statistics (version 19; Armonk, NY). CHIKV infection and dissemination rates were compared with Fisher's exact test with two-tailed *P* values. Non-parametric statistical, Mann–Whitney *U* tests was used to assess the statistical differences for CHIKV titer in w+ and w− groups, and *P* values < 0.05 were considered statistically significant. *P* values were adjusted for multiple tests using the Kruskal–Wallis test with Bonferroni correction.

## Results

### Distribution of *Wolbachia* in Malaysian *Ae. albopictus*.

**Figure 2.**
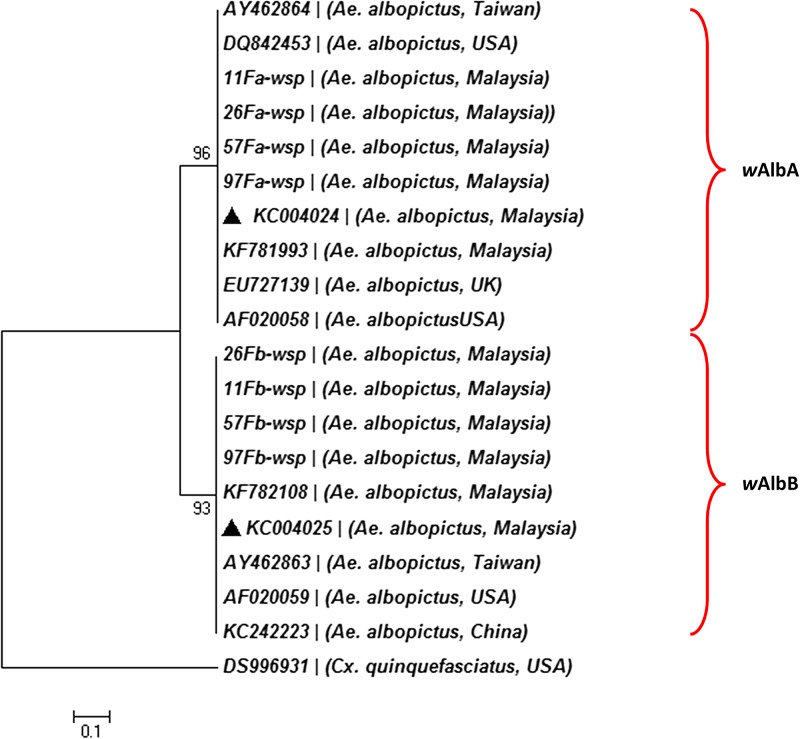
Neighbor-joining phylogenetic tree of *Wolbachia* strain, isolated from *Aedes albopictus* based on partial sequence of *wsp* gene using Kimura-2P analysis. GenBank sequences are shown with accession number. The new sequences of *w*AlbA and *w*AlbB from *Ae. albopictus* obtained in this study were deposited in GenBank with accession nos. KC004024 and KC004025 (indicated by triangle).

A total of 244 *Ae. albopictus* samples collected from eight sites in five states (Malacca, Selangor, Terengganu, Perak, and Pahang) in Malaysia were screened for *Wolbachia* infection using *w*AlbA- and *w*AlbB-specific *wsp* gene primers. Our results showed a high percentage of *Wolbachia* infection with 98.6% in females and 95.1% in males. For *wsp* gene, phylogenetic analysis revealed that *Wolbachia* isolates from the present study were closely related to *Wolbachia* isolates from different geographical regions, and the sequences were grouped into *w*AlbA and *w*AlbB clades, respectively (Figure 2

). The size of the *wsp* fragment for wAlbA was 341 bp and *w*AlbB was 463 bp.

Table 1 shows the frequency of double and single infections of *Wolbachia* in the *Ae. albopictus* populations sampled from all the collection sites. For females, 97.2% (138/142) of the samples were superinfected with both *w*AlbA and *w*AlbB. Only 1.4% (2/142) of samples were singly infected with either *w*AlbA or *w*AlbB. Another 1.4% (2/142) samples were scored as uninfected. For males, 49.0% (50/102) was superinfected with *w*AlbA and *w*AlbB followed by 46.1% (47/102) infection with *w*AlbB only. The remaining 4.9% (5/102) samples were uninfected. None of the males were positive for *w*AlbA only. The uninfected samples were confirmed by running the DNA with the 12S RNA primer set for mitochondrial DNA as a quality check.

### Analysis of linearity and LOD determination.

The linear dynamic range for the multiplex RT-PCR assay was 100% at the range of 100 to 10^2^ RNA copies per reaction but decreased to 88.8% at 10 copies. The LOD was set at 100 copies, which correspond to the mean Cq of 28. Samples with Cq value > 28 was scored as uninfected. Only mosquitoes that score above the LOD was reported to infer CHIKV infection and dissemination in midguts and salivary glands, respectively.

### *Wolbachia* infection status in ovaries of mosquitoes used in the experimental oral infection study.

**Figure 3.**
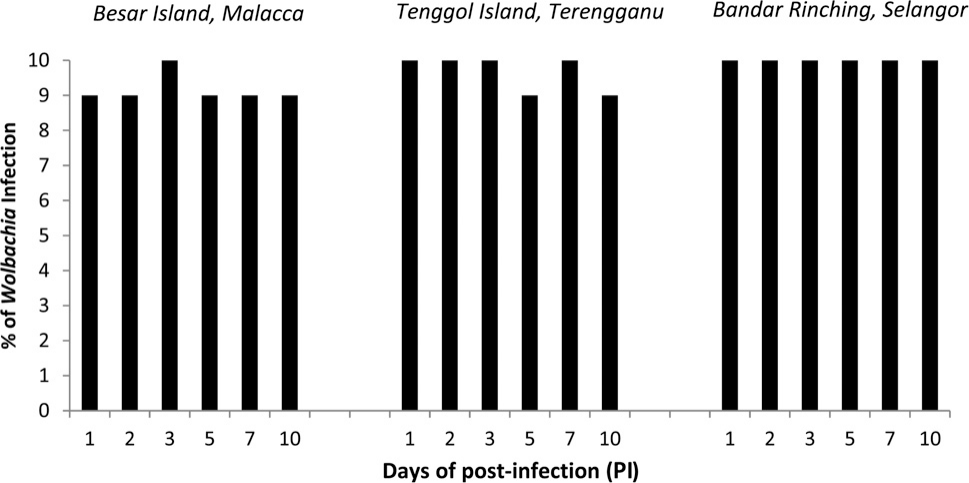
The percentage of *Wolbachia* infection status in ovaries.

The presence of *Wolbachia* in females used in the experimental oral infection study was confirmed by screening the ovaries. It was noticed that ovaries for females were highly infected with at least 90% infection and above. There was no significant difference in the percentage of *Wolbachia* infection for all the time points for the three localities (*P* > 0.05, Fisher's exact test) (Figure 3

).

### Laboratory infection of *Wolbachia* infected (w+) and uninfected (w−) *Ae. albopictus* with CHIKV.

**Figure 4.**
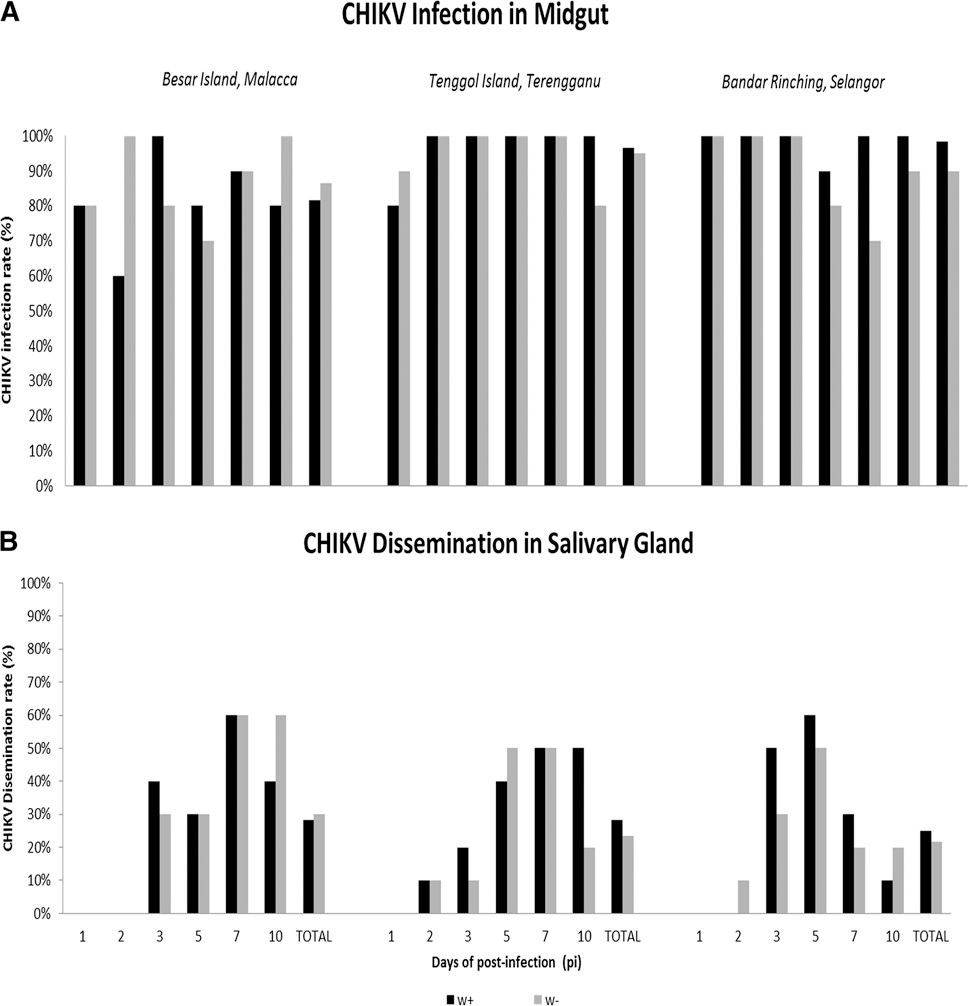
The percentage of chikungunya virus (CHIKV) infection in (**A**) midguts and (**B**) salivary glands for *Wolbachia* infected (w+) and *Wolbachia*-free (w−) for all localities.

CHIKV midgut infections were observed for w+ and w− group for each time point, the midgut infection rate was consistent with percentage of at least 60% for w+ and 70% for w− for all localities (Figure 4A

). There was no significant difference in the infection and dissemination rate between w+ and w− groups for all the time points tested for the three localities (*P* > 0.05, Fisher's exact test). It was noticed that CHIKV was detected in salivary glands as early as day 2 PI (Figure 4B).

**Figure 5.**
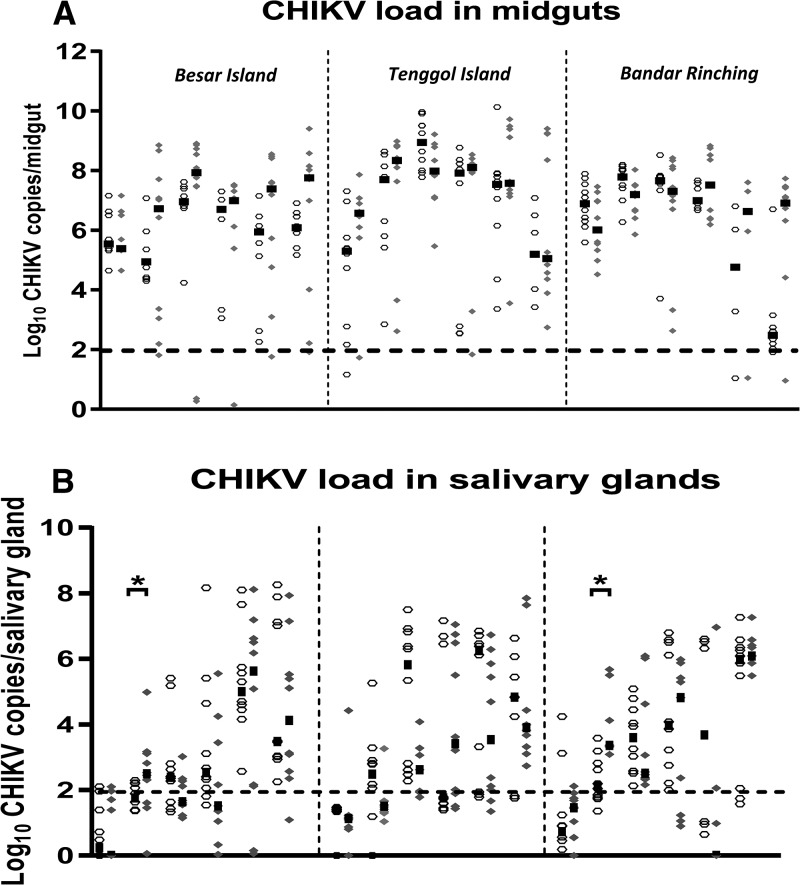
Chikungunya virus (CHIKV) titer in (**A**) midguts and (**B**) salivary glands. Each symbol depicts a CHIKV load from individual organ. At each time point, 8–10 mosquitoes were killed for RNA extraction. Line indicates the median. Dotted line represents the limit of detection.

The number of CHIKV genome copies in midguts was not significantly different between w+ and w− groups at any time point tested. CHIKV titer reached the peak as early as day 2 or day 3. For Besar Island, the peak titer for w+ and w− groups (median = 10^6.9^ versus 10^7.9^ viral copies/midgut, *P* = 0.05) achieved at day 3 PI. For Tenggol Island, the virus replication reached its peak at day 3 for w+ (median = 10^8.9^ viral copies/midgut) and day 2 PI for w− (median = 10^8.3^ viral copies/midgut), respectively. For mosquitoes sampled from Bandar Rinching, the viral copies were at the peak of viral infection for w+ and w− at day 3 PI (median = 10^7.8^ versus 10^7.2^ copies/midgut, *P* = 0.143) (Figure 5A

).

For salivary glands, the amount of CHIKV copies ranged from below the LOD (100 CHIKV copies) to 10^8^ CHIKV copies in both w+ and w− groups. Although there was a variation in median CHIKV titer among the groups, these differences were not statistically significant at other time points, except for day 2 PI (Figure 5B). For Besar Island, CHIKV copy number of w+ group was almost one log lower than for w− group for salivary glands (medians = 10^1.8^ versus 10^2.4^, *P* < 0.05, Mann–Whitney *U* tests) (Figure 5B). Similarly, for Bandar Rinching, CHIKV copies in salivary glands for w+ was lower than w− group (median = 10^2.1^ versus 10^3.3^, *P* < 0.001, Mann–Whitney *U* tests) (Figure 5B).

The total CHIKV titer in different organs was compared between localities as shown in Table 2. The statistical analysis using Kruskal–Wallis test showed that total CHIKV titer in midguts were significantly different from at least one locality (*P* < 0.05). Subsequent post hoc analysis using the Bonferroni correction substantiated the difference. For w+, a significantly lower overall CHIKV titer in midguts was observed in Besar Island compared with Bandar Rinching and Tenggol Island. For w− group, a significantly higher overall CHIKV titer in midguts was observed in Tenggol Island compared with Bandar Rinching and Besar Island. No significant difference for CHIKV titer in salivary glands was observed among the three localities for w+ (*P* = 0.805) and w− (*P* = 0.431).

## Discussion

Our study on the distribution of *Wolbachia* in the field-collected *Ae. albopictus* showed a high infection rate of 98.6% in females and 95.1% in males. Females showed a common superinfection of *w*AlbA and *w*AlbB, occurred at a high prevalence of 97.2%. This is in accordance to other studies, showing superinfection percentage is common in field-collected *Ae. albopictus* infecting at least 96% of mosquitoes.^35^–^39^ *Wolbachia* are expected to rapidly spread to fixation once a *Wolbachia* infection enters a population.^40^,^41^ As females that carry both *w*AlbA and *w*AlbB strain can successfully mate with males that are either singly or superinfected, the superinfection of *Wolbachia* in females may explain the high fidelity of maternal transmission of *Wolbachia* of the mosquito species in the wild.^42^ The males were also most commonly superinfected with *w*AlbA and *w*AlbB at a percentage of 49.0% followed by 46.1% of *w*AlbB-only infection. No single infection of *w*AlbA was detected in males. Apart from the maternal transmission efficacy of *Wolbachia*, the geographical populations are reported to be strong predictors affecting *Wolbachia* infection rate and pattern.^43^ For example, a single-strain (*w*AlbA) infected population has been described in Koh Samui and Mauritius Islands that are geographically distinct from the superinfected populations.^39^,^44^,^45^ A study by Tortosa and others demonstrated that the density of *w*AlbA significantly decreased with age in male *Ae. albopictus* population in which a complete loss was observed within 5-day period postemergence.^46^ Therefore, this finding may serve as a plausible explanation for the lack of single *w*AlbA strain infection in the males despite having females that were superinfected with both *w*AlbA and *w*AlbB strain in the same population. The small percentage of uninfected samples may be due to *Wolbachia* leakage possibly related to the environmental factors such as high temperature and the effect of overcrowding during developmental stage of the larvae, which have been associated with reduced transmission of Wolbachia.^47^

The speculative assumption that native *Wolbachia* might affect the vectorial competence of the mosquitoes was further analyzed by investigating the percentage of CHIKV infection in midguts and dissemination to salivary glands. The artificial oral infection study was conducted in mosquitoes sampled from different geographical regions as genetic susceptibility of *Ae. albopictus* to CHIKV may vary by geography.^48^,^49^ In this study, removing *Wolbachia* did not induce any significant changes of mosquito response to infection by CHIKV. CHIKV was detected in midguts at a high rate of at least 60% and 70% for w+ and w−, respectively, regardless of the population tested. We measured CHIKV dissemination to secondary organs (salivary glands). CHIKV was detected as early as day 2 pi for both w+ and w−, suggesting a short extrinsic CHIKV incubation regardless the presence of *Wolbachia*. This finding is in line with other studies that demonstrated a short CHIKV incubation period of 2 days.^50^,^51^ We also demonstrated that the total CHIKV titer, but not infection susceptibility, is statistically affected by the geographical regions as previously reported.^48^

In this study, CHIKV can massively proliferate in midguts of w+ and w− groups. No difference in CHIKV titer was observed between w+ and w− groups for all time points except for day 2 PI for Besar Island and Bandar Rinching (salivary glands only). As CHIKV ingested by the mosquitoes must pass through the epithelium of the mosquito midgut before infecting salivary gland and other secondary organs, the occurrence of a midgut escape barrier in w+ group can be suggested, limiting the infection of salivary glands. However, as CHIKV inhibition effect was only observed at day 2 PI, it possibly explained a potentially weak midgut barrier caused by *Wolbachia* in its native hosts. Probably, the protection is caused by resource competition between *Wolbachia* and CHIKV in tissue in which they coexists. The presence of *Wolbachia* might limit the availability of resources that are important to ensure achievement of the viral cycle.^23^,^52^–^54^

The higher *Wolbachia* density confers a better protection toward viruses.^55^,^56^ However, the density of native *Wolbachia* in *Ae. albopictus* showed a high level of variation, whether it was from field- or laboratory-established populations.^23^,^35^ Additional studies to see correlation between virus concentration and *Wolbachia* density may provide a better insight to explain the inconsistency in the *Wolbachia*-mediated virus replication in the oral-challenge experiments in this study. However, the detection of the RNA genome only did not give definitive evidence of the viability of the virus. For example, Wong and others in the CHIKV oral-challenged in *Ae. aegypti*, wherein a persistent CHIKV RNA detection was reported in the mosquito eggs and adults progeny. However, it was not proven to be viable and infectious virus, omitting the possibility of the vertical transmission for CHIKV in *Aedes* sp.^57^ Nonetheless, we cannot exclude the limitation for using the quantitative PCR for virus detection as the RNA viral copy number can give an overestimation of infectious viral particles. Given the high sensitivity of real-time PCR in detecting the region encoding for E1 protein of CHIKV,^57^ even a slight contamination of virus nucleic acid on the surface of the organs (from tissue beside the organs) would have been sufficient to prime the RT-PCR reaction and hence yielding the overestimation of the infection percentage in the secondary organs including salivary glands.

## Conclusion

Our results suggest a high prevalence of *Wolbachia* infection in the wild-caught *Ae. albopictus*. In accordance to other studies, the *Ae. albopictus* are naturally infected with *w*AlbA and *w*AlbB. Our data showed that the presence of *Wolbachia* do not pose any significant impact in the CHIKV infection in the midguts and dissemination to salivary glands in its native host, *Ae. albopictus*. The presence of *Wolbachia* does not interfere with the extensive CHIKV replication in midguts. Nonetheless, the native *Wolbachia* has a minimal effect on the CHIKV titer in the salivary glands, explaining why *Ae. albopictus* is a competent vector for CHIKV despite naturally infected with *Wolbachia*.

## Figures and Tables

**Table 1:** *Wolbachia* in individual field-collected *Aedes albopictus* from various collection sites

Study sites	Types of habitat	Total female	Infected %	Infected female % (*n*)				Total Male	Infected %	Infected male % (*n*)			
				*w*AlbA and *w*AlbB	*w*AlbA	*w*AlbB	Uninfected			*w*AlbA and *w*AlbB	*w*AlbA	*w*AlbB	Uninfected
Bukit Beruang, Malacca	Residential area	13	100	100 (13)	0	0	0	2	100	100 (2)	0	0	0
Besar Island, Malacca	Island	26	100	100 (26)	0	0	0	18	100	50 (9)	0	50 (9)	0
Ketam Island, Selangor	Island	22	100	100 (22)	0	0	0	5	100	0	0	100 (5)	0
Carey Island, Selangor	Plantation	4	100	100 (4)	0	0	0	5	100	20 (1)	0	80 (4)	0
Kajang, Selangor	Residential area	12	100	100 (12)	0	0	0	15	100	6.7 (1)	0	93.3 (14)	0
Marang, Terengganu	Seashore	17	100	100 (17)	0	0	0	19	100	57.9 (11)	0	42.1 (8)	0
Pangkor Island, Perak	Island	32	93.7	87.5 (28)	3.1 (1)	3.1 (1)	6.3 (2)	22	86.4	54.6 (12)	0	31.8 (7)	13.6 (3)
Kuantan, Pahang	Plantation	16	100	100 (16)	0	0	0	16	87.5	87.5 (14)	0	0	12.5 (2)
TOTAL		142	98.6	97.2 (138)	0.7 (1)	0.7 (1)	1.4 (2)	102	95.1	49.0 (50)	0	46.1 (47)	4.9 (5)

**Table 2:** Median CHIKV copy number (IQR) of total CHIKV titer in midguts and salivary glands among localities for w+ and w− groups

Locality	Sample size		CHIKV titer Median copy number (IQR)			
			w+		w−	
	w+	w−	Midguts	Salivary glands	Midguts	Salivary glands
Besar Island, Malacca	55	59	6.07 (5.21, 6.93)^a^*	2.38 (1.63, 4.63)^a^	7.02 (4.01, 7.80)^a^	2.16 (1.22, 3.45)^a^
Tenggol Island, Terengganu	58	60	7.44 (5.20, 8.09)^b^	2.70 (1.45, 6.36)^a^	7.83 (6.10, 8.53)^b^	2.25 (1.44, 4.34)^a^
Bandar Rinching, Selangor	60	57	7.01 (6.04, 7.69)^b^	2.93 (1.73, 5.39)^a^	6.98 (6.02, 7.50)^a^	2.81 (1.28, 5.50)^a^

Data are presented as median CHIKV copy number (IQR) and were compared among localities by Kruskal–Wallis test and Mann–Whitney *U* test for post hoc pairwise comparisons. CHIKV = chikungunya virus; IQR = interquartile range.

* Median with different superscripts (i.e., a and b) within the same column indicates significant difference (*P* < 0.05).
